# KmerGO: A Tool to Identify Group-Specific Sequences With *k*-mers

**DOI:** 10.3389/fmicb.2020.02067

**Published:** 2020-08-25

**Authors:** Ying Wang, Qi Chen, Chao Deng, Yiluan Zheng, Fengzhu Sun

**Affiliations:** ^1^Department of Automation, Xiamen University, Xiamen, China; ^2^Xiamen Key Laboratory of Big Data Intelligent Analysis and Decision-Making, Xiamen, China; ^3^Quantitative and Computational Biology Program, Department of Biological Sciences, University of Southern California, Los Angeles, CA, United States

**Keywords:** group-specific *k*-mer, sequences comparison, high-throughput sequencing data, genomic comparison, metagenomic comparison

## Abstract

Capturing group-specific sequences between two groups of genomic/metagenomic sequences is critical for the follow-up identifications of singular nucleotide variants (SNVs), gene families, microbial species or other elements associated with each group. A sequence that is present, or rich, in one group, but absent, or scarce, in another group is considered a “group-specific” sequence in our study. We developed a user-friendly tool, KmerGO, to identify group-specific sequences between two groups of genomic/metagenomic long sequences or high-throughput sequencing datasets. Compared with other tools, KmerGO captures group-specific *k*-mers (*k* up to 40 bps) with much lower requirements for computing resources in much shorter running time. For a 1.05 TB dataset (.fasta), it takes KmerGO about 21.5 h (including *k*-mer counting) to return assembled group-specific sequences on a regular stand-alone workstation with no more than 1 GB memory. Furthermore, KmerGO can also be applied to capture trait-associated sequences for continuous-trait. Through multi-process parallel computing, KmerGO is implemented with both graphic user interface and command line on Linux and Windows free from any pre-installed supporting environments, packages, and complex configurations. The output group-specific *k*-mers or sequences from KmerGO could be the inputs of other tools for the downstream discovery of biomarkers, such as genetic variants, species, or genes. KmerGO is available at https://github.com/ChnMasterOG/KmerGO.

## Implementation

### Background

Fast developments of high-throughput sequencing technologies spout large volume of shotgun genomic/metagenomic data. The comparisons of high-throughput sequencing data under various phenotypes are critical to understand the mechanism behind their differences.

Short *k*-mer (*k* < 15) based measures, such as $d_{2}^{s}$, $d_{2}^{*}$ and *CVtree*, calculate dissimilarity between sequences or high-throughput sequencing samples (Jiang et al., 2012; Liao et al., 2016; Song et al., 2019) using the global statistical models. Based on long *k*-mers (*k* > 21), Mash (Ondov et al., 2016), Skmer (Sarmashghi et al., 2019), and Kmer-db (Deorowicz et al., 2018) use MinHash to approximate Jaccard distance between pairwise sequences based on randomly sampled small set of *k*-mers. However, these measures only return dissimilarity between two data sets, but do not capture specific biomarkers associated with different phenotypes.

Long *k*-mers contain richer biological information and are able to depict specific signatures in nucleotide sequences (Wang et al., 2016). Therefore, *k*-mers with length ≥20 bp have been utilized to identify biomarkers, such as sequences (Drouin et al., 2016; Wang et al., 2018), genetic variants (Jaillard et al., 2018; Rahman et al., 2018; Standage et al., 2019), and genes (Han et al., 2017) specific to categorical phenotypes. Rahman et al. (2018) identified the significant differentially abundant *31*-mers between two human populations and then discovered single nucleotide polymorphisms (SNPs). Two long *k*-mer based GWAS tools were developed for bacterial genomes to detect *de Novo* variants (Jaillard et al., 2018; Standage et al., 2019). For microbial community, Han et al. (2017) predicted microbial genes in the gut associated with type II diabetes (T2D) by detecting differentially abundant *21*-mers. In our previous study, we developed a computational framework using *40*-mers (Wang et al., 2018) to capture group-specific sequences between two groups of large-scale metagenomic datasets, including LC (Liver Cirrhosis)-associated (Qin et al., 2014), IBD (Inflammatory Bowel Disease)-associated (Qin et al., 2010) and WT2D (Type 2 Diabetes in Women)-associated (Karlsson et al., 2013). The assembled group-specific sequences possess the discriminative power to separate the samples from disease and health groups.

“Group-specific” means elements (*k*-mers, genes, species, genetic variants) that are present, or rich, in one group, but absent, or scarce, in another group. Specifically, a group-specific *k*-mer in our study means only using the current *k*-mer as a single feature can separate the case and control groups with accuracy higher than a preset threshold. No matter what final group-specific elements are, the identification of group-specific *k*-mers is the common key step. It is also the most consuming step for computing time and resource. However, the tools developed by the studies mentioned above, MetaGO (Wang et al., 2018), HAWK (Rahman et al., 2018), Kover (Drouin et al., 2016) and Kevlar (Standage et al., 2019), required high memory; or/and complex prerequisites of supporting environments, packages; or/and complicated deployments, which are described in detail in Tables 1, 2. KMC3 (Kokot et al., 2017) and GenomeTester4 (Kaplinski et al., 2015) offer set operations for *k*-mers. Our experiments demonstrate that they cannot return *k*-mer frequency matrix and they can only obtain strictly-limited unique *k*-mers that are present in 100% of samples in one group and absent from 100% in the other group using a combination of set operations. However, biological samples are highly diverse, and the strict limitation on unique k-mers would miss some potential useful *k*-mers that have different abundance profiles in two groups or that are present in most samples in one group and absent in most samples in the other group.

**TABLE 1:** Installation and running requirements of the five tools.

	**Final purpose**	**Installation requirements**	**Operation system**	**Running interface**
KmerGO	Group-specific sequences	No prerequisites; No installation; One-click running.	Linux, Windows	Graphic User interface; Command Line
MetaGO (Wang et al., 2018)	Group-specific sequences	Deployment of *Spark*, Python.	Linux	Command Line
HAWK (Rahman et al., 2018)	Group-specific genetic variants	R with *foreach* and *doParallel* packages; JELLYFISH; EIGENSTRAT; ABYSS.	Linux	Command Line
Kover (Drouin et al., 2016)	Group-specific k-mers and then mapped to genes	CMake; GNU C++ compiler; GNU Fortran; The HDF5 library; NumPy; Python 2.7.x; Python development headers; SciPy.	Linux, Mac	Command Line
Kevlar (Standage et al., 2019)	Group-specific genetic variants	Python 3 with *network* and *khmer* packages; *pysam* module; *pandas*, *scipy* and *intervaltree* librarys; BWA.	Linux	Command Line

**TABLE 2:** Testing five tools on two groups of *E. coli* high-throughput sequencing dataset.

	**Memory peak**	Running time*	**Number of group-specific *k*-mers**
KmerGO	305 MB	40 min	1,087 (ASS = 0.8); 6,156 (ASS = 0.7)
MetaGO (Wang et al., 2018)	50 MB	3 h	1,087 (ASS = 0.8); 6,156 (ASS = 0.7)
HAWK (Rahman et al., 2018)	3.91 GB	2.05 h	4,446
Kover (Drouin et al., 2016)	>128 G	In the step *dsk2kover*, Kover was terminated by the workstation	
		because the running required more than 128 GB memory.	
Kevlar (Standage et al., 2019)	76.95 G	In the step *Kevlar novel*, it took Kevlar 6.7 h to process every 5,000,000 reads.	
		Because the total number of reads in testing data is more than 297,000,000,	
		it would require about 400 h to finish the processing. We stopped the experiment.	

*The running time of *k*-mer counting was exclude because they all used different tools of third-party.

Therefore, we developed a tool, KmerGO, to identify group-specific sequences between two groups of sequences or high-throughput sequencing datasets. We also extended KmerGO to capture trait-associated sequences for continuous trait, such as height, weight, blood pressure and so on. KmerGO offers a user-friendly graphical interface with one-click installation free from any configurations. KmerGO is computational efficient running with a loser tree structure and multiple processes with low requirement for memory, which can be run on a regular stand-alone server with Linux or Windows.

### The Framework of KmerGO

**FIGURE 1:**
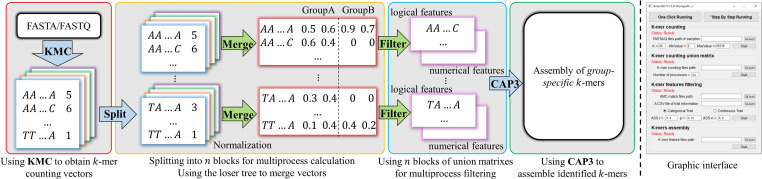
The diagram of KmerGO: KMC3 is adopted to obtain *k*-mer counting vector for each sample. Each vector is split into *n* blocks for calculating the union matrix over two groups of samples and filtering for group-specific *k*-mers in multiple processes. CAP3 is then used to assemble the group-specific *k*-mers into sequences. The right side figure is the graphic interface of KmerGO.

KmerGO is developed by *C++* and *Python*, offering running modes of graphical user interface and command line. As shown in Figure 1, KmerGO includes four modules, producing *k*-mer counting vector, obtaining union of *k*-mer counting vectors over two groups of samples (called “*k*-mer frequency matrix” in our study), identifying group-specific *k*-mers, and assembling group-specific sequences. The modules producing *k*-mer frequency matrix and group-specific *k*-mers are implemented on multiple processes. The graphic interface of KmerGO is shown in the right panel of Figure 1.

#### Mode I: *k*-mer Counting

KMC3 (Kokot et al., 2017) is adopted to count the number of occurrences of each *k*-mer within the sequencing data and takes complementary *k*-mers into consideration. Only the *k*-mers occurred equal or greater than a certain threshold (default is 2) are kept. Then *k*-mers are sorted according to their lexicographic order using KMC3. This module produces a *k*-mer counting vector for each sample data.

#### Mode II: *k*-mer Frequency Matrix

**FIGURE 2:**
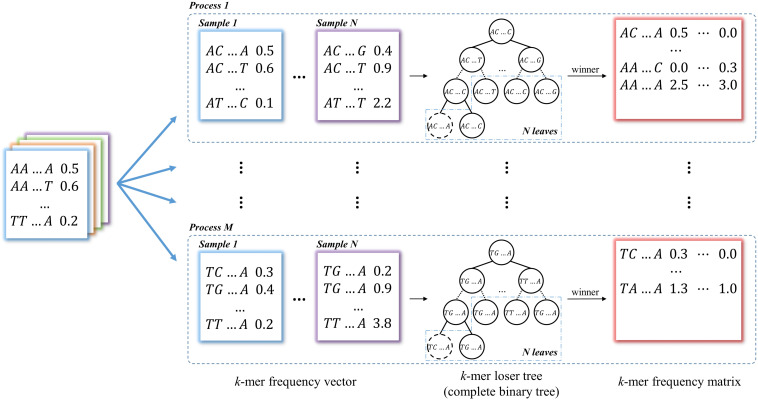
The schematic diagram of producing *k*-mer frequency matrix. The *k*-mer frequency vectors are split into *n* processes (*n* from 1 to 256) based on *k*-mer prefix in lexicographic order. The loser tree is built and updated iteratively based on *N* sample frequency vectors in each process. The winners of loser tree are written to the *k*-mer frequency matrix.

In this module, *k*-mer counting is normalized by the total number of occurrences in the vector for each data. Then all the *k*-mer vectors from two groups of data are merged into a *k*-mer frequency matrix through *union* operations with each *k*-mer as a row and each data as a column, which is used for identifying group-specific *k*-mers. This is the most time-consuming step in most long *k*-mer based tools. In KmerGO, we adopt multi-processes parallel computing and loser tree algorithm to accelerate the running. The schematic diagram is shown in Figure 2. In KmerGO, sorted *k*-mer vectors are split into *n* processes (*n* from 1 to 256) based on *k*-mer prefix in lexicographic order to implement multi-processing parallel computing. For example, when *n* is 4, *k*-mer frequency vectors are split by their initials (A, C, G, or T). The split operation is implemented using jumping files’ pointer on *Multiprocessing* package in *python*. The *k*-mer loser tree is built and updated iteratively in each process to fulfill fast *k*-mer comparison.

##### The description of the loser tree structure

**FIGURE 3:**
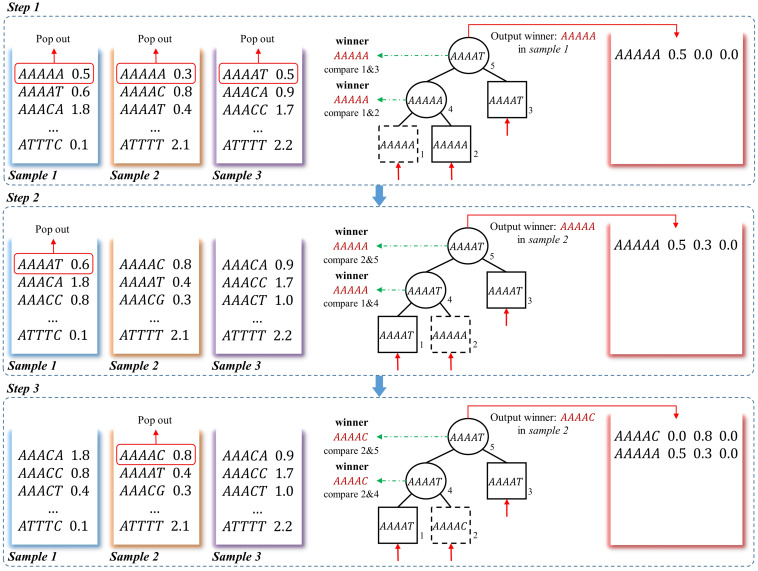
A schematic example to obtain frequency matrix using loser-tree algorithm. Using a three-sample dataset as an example, in step 1, the first *k*-mers of the three frequency vectors are pop-out as the leaves of a loser tree. Because “AAAAA” = “AAAAA,” the “AAAAA” in *sample 1* is randomly picked as the winner and the other one is kept as the loser in the Parent node. The winner “AAAAA” is then compared to another leaf node “AAAAG,” the larger one “AAAAG” is the loser and kept as root node. The winner “AAAAA” and its corresponding frequency in *sample 1* is written to the frequency matrix. In step 2, the second *k*-mer “AAAAT” in *sample 1* is pop-out to replace the previous winner node “AAAAA.” “AAAAT” is compared to Parent node “AAAAA” in second level, and the “AAAAA” in *sample 2* is the winner and the Parent node of this branch is updated as the loser “AAAAT.” Then the winner “AAAAA” is still the winner when compared to root node “AAAAG”, so the corresponding frequency of “AAAAA” in *sample 2* is updated in the frequency matrix. In step 3, the winner is “AAAAC,” which means there is no other samples containing *k*-mer “AAAAA.” And the winner and its corresponding frequency is written to the frequency matrix.

The loser tree is a tournament binary tree (Sahili, 2004), which was originally designed for fast numerical comparison. An example of generating frequency matrix using the loser-tree algorithm is shown in Figure 3. For *N* samples, the first *k*-mer of each vector is read out and build a binary loser-tree with each *k*-mer as a leaf node. Two children nodes are compared, and then the winner (smaller *k*-mer) is pop-out to compare with upper level node and the loser (larger *k*-mer) is kept as parent node. The final winner and its frequency are written to the union frequency matrix. In the following update steps, the previous winner leaf node is replaced by the second *k*-mer from the same sample. The corresponding loser nodes are then updated with hierarchical comparison between the new node with its parent node, then the final winner is written to the frequency matrix. The processing is repeated until all the *k*-mers from all the samples are written to the matrix.

##### The complexity analysis of the loser tree structure

We assume that the number of *k*-mers in each sample is *M* and the number of samples is *N*. For *k = 40*, *M* is between 10^8^ and 10^9^. (1) Loser tree structure has significant superiority in space complexity. In each round of iteration, loser tree reads one *k*-mer from each sample, and stores *k*-mers using a binary tree structure, so totally 2*N k*-mers in memory. Therefore, the space complexity *S*(*M*,*N*) = *O*(*N*), which is the reason that the peak memory of KmerGO is only about 300 MB for Tera Bytes of dataset. In comparison, GenomeTester4 obtains the *k*-mer union set on groups of samples using pair by pair union operations. To avoid frequent hard-disk reading-writing operations, the files necessary for the following iterations should be kept in memory. Therefore, the space complexity of pairwise union algorithm is *S*(*M*,*N*) = *O*(*MN*), where the number of *k*-mer_*s*_ *M* increases exponentially with the growth of *k*-mer length *k*. In addition, if we want to reduce the space complexity from *O*(*MN*) to μ*O*(*MN*) (0 < μ < 1), the hard-disk reading-writing time complexity will increase from *O*(*MN*) to *O*(*MN* + (_log2_*N*−1)(1−μ)*MN*). (2) The time complexity of the loser tree in KmerGO is *T*(*M*,*N*) = *O*(*MN*_log2_*N*). Because in each iteration, when a new *k*-mer replaces the pop-out node in the last iteration of the existing loser tree, the new *k*-mer is only required to compare with its parent node hierarchically, so only _log2_*N* comparisons are required. However, if the new *k*-mer is directly compared to the remaining (*N*−1) *k*-mers to find the smallest *k*-mer instead of using the loser tree, the time complexity of comparison is *O*(*N*). Thus, the overall time complexity would be *T*(*M*,*N*) = *O*(*MN*^2^), which is larger than *T*(*M*,*N*) = *O*(*MN*_log2_*N*) in the loser tree.

Therefore, loser tree is better than pairwise union strategy in space complexity; and is better than direct sorting among all the samples in time complexity. Furthermore, if the final winner *k*-mer is different from the winner of the previous iteration, the union of the current *k*-mer is complete, which does not require traverse all the samples. Once the loser tree is built for the first *k*-mers from the *N* samples, it is only required to update the corresponding nodes for the new incoming *k*-mer.

#### Mode III: Group-Specific *k*-mer Identification

In the module of group-specific *k*-mers, the *k-*mers absent in more than 80% of control samples and 80% of case samples are removed. KmerGO uses the strategy from MetaGO (Wang et al., 2018) to identify group-specific *k*-mers, because the performance of the strategy has been evaluated and validated by that study. The processing strategy are briefly described as follows. The group-specific *k*-mers are obtained using the following criteria: (1) If the average of sensitivity and specificity (ASS) for classifying cases versus controls using the current single *k*-mer’s presence or absence in the sequencing data is higher than a preset threshold, the *k*-mer is considered as group-specific; (2) If the difference of the current *k*-mer’s frequencies between two groups are statistically significant with *p*-value less than a preset threshold (e.g., 0.05) based on the *Wilcoxon rank sum test* and the ASS is higher than a preset threshold using logistic regression, the *k*-mer is considered as group-specific. The detail descriptions about the identification for the group-specific *k*-mers can be found in Section 2 of MetaGO (Wang et al., 2018).

Furthermore, KmerGO is extended to capture trait-associated sequences for dataset with continuous trait. The processing strategy is also composed of two parts: (1) For presence/absence of a *k*-mer, we compare the distributions of trait values of individuals having the *k*-mer with that of individuals not having the *k*-mer using the *Wilcoxon rank sum test.* The *k*-mer is considered as trait-associated if the resulting *p*-value is less than a preset threshold; (2) For *k*-mer abundance, we calculate the *Spearman’s rank correlation coefficient* between the current *k*-mer’s frequencies and the trait values of the samples. If the correlation coefficient is higher than a preset threshold, the *k*-mer is considered as trait-associated.

#### Mode IV: Identifying Group-Specific Sequences Through Group-Specific *k*-mer Assembly

In the module of group-specific sequences assembly, KmerGO uses CAP3 (Huang and Madan, 1999) to assemble the identified group-specific *k*-mers into sequences. Only using the overlap information between *k*-mers, the specific parameter settings for CAP3 is shown in the Supplementary Material.

## The Functions of KmerGO

KmerGO supports end-to-end running or mid-way input and output. Therefore, KmerGO can be used in the following three situations:

• Identify group-specific/trait-associated *k*-mers/sequences from categorical- or continuous- trait based on sequences or high-throughput sequencing datasets. The group-specific/trait-associated *k-*mers/sequences can be used for the follow-up discovery of biomarkers, such as genetic variants, species, or genes.
• Obtain union matrix of *k*-mer frequency vectors from multiple high-throughput sequencing data or multiple files with long sequences, where the input could be sequencing files (.fasta,.fastq, fasta.gz, fastq.gz) or frequency vectors with text format from the KMC tool. Users can also run KmerGO for union matrix and then make the filtering for group-specific *k*-mers with their own strategies.
• Output group-specific elements for a matrix composed of the features from two groups of samples. The features could be the abundances, frequencies or other quantified features.

## Comparison With Other Four Tools in Identifying Group-Specific *k*-mers

Although MetaGO (Wang et al., 2018), HAWK (Rahman et al., 2018), Kover (Drouin et al., 2016), and Kevlar (Standage et al., 2019) were designed for identifying different group-specific elements, all of them include the key step of identify group-specific *k*-mers. Therefore, KmerGO and the four tools were installed and applied to a testing data for comparison. The testing data is from the testing experiment of HAWK (Rahman et al., 2018). We installed and ran the five tools in a stand-alone workstation in Linux.

### Installations and Running Requirements

KmerGO is free from any installation and environmental configuration. It is run directly with the executive file. The other four tools have different prerequisites of supporting environments, packages, or/and complicated deployments. KmerGO is the only one to offer GUI (Graphic User Interface) among the five tools. The detail of installation and running requirements are shown in Table 1.

### Experiments on a Testing Dataset

The testing dataset composes of 241 high-throughput sequencing data of *Escherichia coli* strains. The dataset had been used to test the performance of HAWK (Rahman et al., 2018). The two groups are 189 *E. coli* strains resistant to ampicillin and 52 *E. coli* strains sensitive to ampicillin. The size of the dataset is 116 GB in.fasta format. The five tools were applied to the testing dataset to identify the group-specific *31*-mers in the workstation with regular configuration of *Intel Xeon E5-2620 v4* (2.10 GHz, 8 cores, 16 threads) and 128 GB memory. We set *k* = 31 because 31 is the default setting for most of the tools. When recording the running time, we excluded the *k*-mer counting step (because different third-party tools were integrated) and the steps after the identification of group-specific *k*-mers. On our testing workstation, only KmerGO, MetaGO, and HAWK successfully finished the running on the testing data and output group-specific *k*-mers. As shown in Table 2, it takes KmerGO 40 min with only 305 M peak memory for the 116 GB dataset. By contrast, it takes HAWK 2 h with 3.91 GB peak memory. Although the peak memory of MetaGO is only 50 MB, the running time is 3 h, longer than KmerGO and HAWK. The running descriptions of Kover, Kevlar are given in Table 2.

### Comparison of Group-Specific *k*-Mers Identified by KmerGO and HAWK

**FIGURE 4:**
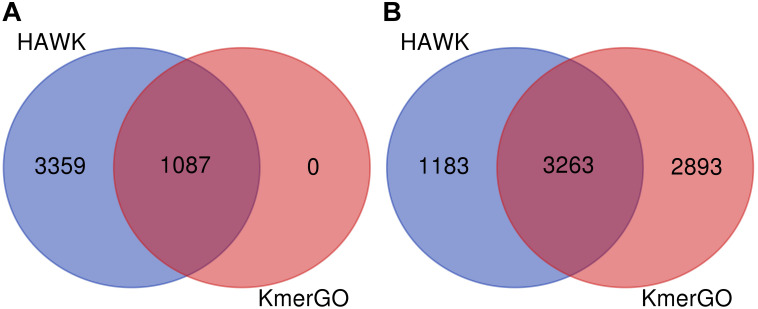
The Venn diagram of group-specific *k*-mers identified by HAWK and KmerGO with **(A)** ASS = 0.8 and **(B)** ASS = 0.7.

Because KmerGO adopted the identical filtering strategy for group-specific *k*-mers with MetaGO, their output results are the same. We compared the group-specific *k*-mers identified by KmerGO and HAWK. The dataset are two groups of *E. coli* strains resistant and sensitive to ampicillin, respectively. As shown in Figure 4, when ASS threshold is set as 0.8, KmerGO identified 1,087 resistant-specific *31*-mers, and all of them are included in the 4,446 resistant-specific *31*-mers by HAWK. When ASS threshold is relaxed to 0.7, KmerGO output 6,156 resistant-specific *31*-mers, and 3,263 of them overlap with results of HAWK. Both KmerGO and HAWK do not find any sensitive-specific *31*-mers. This result is consistent with the analysis of the original paper (Earle et al., 2016) of the dataset, which mentioned the resistance mechanism is caused by the presence of β−*lactamasegenesbla*_*TEM*_. Therefore, no control associated (sensitive-specific) markers would be found (Earle et al., 2016). The difference of the identified group-specific *k*-mers between KmerGO and HAWK is because they used various filtering strategies. The objective of HAWK is to find SNPs from a single genome that distinguish cases from controls. HAWK computes *p*-values using likelihood test assuming Poisson distributions for the numbers of occurrences of *k*-mers in both cases and controls, and then adjusts *p*-values based on the first ten principal components of the numbers of occurrences of *k*-mers to correct for population stratification. KmerGO outputs group-specific *k*-mers, and each of them has distinguishing power to separate two groups. Therefore, different objectives of the two tools lead to differences of their results.

Compared with MetaGO (Wang et al., 2018), HAWK (Rahman et al., 2018), Kover (Drouin et al., 2016), and Kevlar (Standage et al., 2019), KmerGO has several advantages: (1) KmerGO can be run with one-click installation under Windows and Linux operating systems, free from any environmental configurations and deployments. (2) KmerGO offers both graphic user interface and command lines, which supports the easy running for biologists and HPC job submission. (3) KmerGO is faster than other tools with much lower memory requirements. (4) KmerGO is applicable to handle both genomic and metagenomic data, long sequences and high-throughput sequencing data.

## Comparison With KMC3 and Genometester4: Two Additional Tools to Identify Unique *k*-mers

KMC3 (Kokot et al., 2017) and GenomeTester4 (Kaplinski et al., 2015) integrated the *k*-mer counting and set operations. According to their available options, KMC3 and GenomeTester4 cannot obtain the *k*-mer frequency matrix for multiple *k*-mer vectors. Instead, they can only output the *k*-mer union set and the corresponding sum/min/max of their frequencies for multiple *k*-mer frequency vectors due to their processing data structures.

Furthermore, KMC3 and GenomeTester4 can only strictly-limited unique k-mers. The difference between a unique and a group-specific *k*-mer is that the unique *k*-mers are required to be present to all the samples of one group but absent from all the samples of another group. When the threshold ASS = 1, a group-specific *k*-mer is a unique *k*-mer. Therefore, the set of unique *k*-mers is the special case of the set of group-specific *k*-mers.

The basic idea of KMC3 and GenomeTester4 to obtain unique *k*-mers can be described as follows. Let as *A*_*i*_ and *B*_*j*_ be the numbers of occurrences of a certain *k*-mer in sample *i* of group A and sample *j* of group B, respectively. If $\sum_{i\in A}A_{i}+\sum_{j\in B}B_{j}=\sum_{i\in A}A_{i}$, the current *k*-mer is unique to group A; If $\sum_{i\in A}A_{i}+\sum_{j\in B}B_{j}=\sum_{j\in B}B_{j}$, the current *k*-mer is unique to group B. In KMC3, the idea is implemented by the combination of “*kmc_tools*,” “*union*,” “*intersection*,” “*counters_subtract*,” “*kmers_subtract.*” In GenomeTester4, the idea is implemented by “*glistcompare*,” “*union*,” *“intersection,” “diff_union.”* The running scripts of KMC3 and GenomeTester4 are available at Supplementary Material.

KMC3 and GenomeTester4 are also applied to the 241 high-throughput sequencing data of *Escherichia coli* strains that are tested on the five tools in last section. Compared with the 6,156 and 4,446 resistant-specific *k*-mer identified by KmerGO and HAWK, KMC3 and GenomeTester4 do not find any unique *k*-mer to resistant group. The experiment demonstrates that KMC3 and GenomeTester4 can only implement highly inflexible filtering. However, biological individuals are highly diverse, the strict limitation would miss potential useful k-mers having consistent characteristics in most cases instead of all cases.

## Application of KmerGO on a Large-Scale Metagenomic Sequencing Dataset

KmerGO was also applied to the large-scale metagenomic liver cirrhosis-associated dataset (Qin et al., 2014), which was tested on MetaGO (Wang et al., 2018). The dataset includes 66 liver cirrhosis patients and 56 healthy controls with high-throughput sequences by Illumina HiSeq 2000 with 1.07 TB file size in.fasta format. Using the regular stand-alone workstation with CPU *Intel(R) Xeon(R) E5-2620 v4* and 128G memory for *k* = 40, it takes 21.5 h to identify the group-specific sequences, including 4 h *k*-mer counting by KMC3 and 17.5 h (16 processes) for obtaining the union matrix and identifying the group-specific *k*-mers. The memory peak is no more than 1 GB. The output of KmerGO is identical with that of MetaGO, and the effectiveness, excellent performance and biological implications were validated in the MetaGO paper (Wang et al., 2018).

## Conclusion

Group-specific nucleotide sequences offer important information to understand the differences between two groups of genomic/metagenomic samples. Free from reference sequences, assembly, and alignment, KmerGO identifies group-specific/trait-associated sequences (*k* up to 40 bps) and return the assembled group-specific/trait-associated sequences. The identified *k*-mers present discriminant power, paving the way for a new paradigm of biomarker discovery for different phenotypes.

Free from any pre-installed supporting environments, packages, and complex configurations, KmerGO offers a graphic user interface by direct running the executive file for Linux and Windows, and command line running mode for job submission in HPC, which is extraordinary friendly to users from various backgrounds. Through multi-processing parallel computing, KmerGO is highly time efficient with low requirements for computational resources (CPU, memory). Therefore, on a regular standalone workstation, it takes KmerGO a total of 21.5 h to output group-specific *k*-mers for 1.05 TB (.fasta) two groups of high-throughput sequencing data.

KmerGO is suitable for both long sequences and high-throughput sequencing data. Supporting end-to-end running or mid-way input and output, KmerGO can also be a tool to obtain the union matrix over *k*-mer frequency vector of a large number of samples; to filter the group-specific elements for feature matrix composed of two groups of samples with quantified values. The output group-specific *k*-mers or sequences from KmerGO could be the input of other tools for the following discovery of biomarkers, such as genetic variants, species, or genes.

## Data Availability Statement

All datasets presented in this study are included in the article/Supplementary Material.

## Author Contributions

YW and FS designed the KmerGO. QC developed and implemented the KmerGO. CD and YZ proposed the algorithms for KmerGO. All authors read and approved the final manuscript.

## Conflict of Interest

The authors declare that the research was conducted in the absence of any commercial or financial relationships that could be construed as a potential conflict of interest.
