# Supplementary material for: KmerGO: A Tool to Identify Group-Specific Sequences With k-mers
Source: Front Microbiol. 2020 Aug 25;11:2067. doi: 10.3389/fmicb.2020.02067 (PMC7477287; doi:10.3389/fmicb.2020.02067)
Supplement: Supplementary file 1 [file Table_1.DOCX]

***Supplementary Note 1***

1 KMC3 (version 3.0.0) running command to identify unique k-mers

1.1 Folders

1.1.1 Files in KMC3 program files folder

| Name | Type | Description |
| --- | --- | --- |
| groupA | Folder | The result of group A |
| groupB | Folder | The result of group B |
| ALL.txt | Text | All [high-throughput](javascript:;) [sequencing](javascript:;) file paths |
| GroupA.txt | Text | All group A [high-throughput](javascript:;) [sequencing](javascript:;) file paths |
| GroupB.txt | Text | All group B [high-throughput](javascript:;) [sequencing](javascript:;) file paths |
| run.sh | Linux shell script | A script to run this program |

1.1.2 Files in *groupA* folder

| Name | Type | Description |
| --- | --- | --- |
| complex.op | KMC3 complex operation file | Intersection operation of group A |
| count.sh | Linux shell script | A script to count group A k-mers |

1.1.3 Files in *groupB* folder

| Name | Type | Description |
| --- | --- | --- |
| complex.op | KMC3 complex operation file | Intersection operation of group B |
| count.sh | Linux shell script | A script to count group B k-mers |

1.2 Files

1.2.1 File *run.sh*

| #!/bin/bash  kmc=kmc  kmc_tools=kmc_tools  kmc_dump=kmc_dump  t=20  k=31  mkdir -p tmp  /usr/bin/time -v taskset -c 0-$(($t-1)) $kmc -t$t -k$k -ci2 -cs16777215 -fm @ALL.txt ALL_kmc2 tmp &> count_ALL.log  /usr/bin/time -v taskset -c 0-$(($t-1)) $kmc_tools transform ALL_kmc2 sort ALL &> optimize_DB.log  (cd groupA;/usr/bin/time -v taskset -c 0-$(($t-1)) ./count.sh $kmc $k $t &> count.log)  (cd groupA;/usr/bin/time -v taskset -c 0-$(($t-1)) $kmc_tools -hp -t$t complex complex.op &> intersection.log)  (cd groupA;/usr/bin/time -v taskset -c 0-$(($t-1)) $kmc_tools -hp -t$t simple A_intersection ../ALL intersect inter2 -ocmax &> ALL_intersect.log)  (cd groupA;/usr/bin/time -v taskset -c 0-$(($t-1)) $kmc_tools -hp -t$t simple inter2 A_intersection counters_subtract invalid &> counters_subtract.log)  (cd groupA;/usr/bin/time -v taskset -c 0-$(($t-1)) $kmc_tools -hp -t$t simple A_intersection invalid kmers_subtract A_specific &> kmers_subtract.log)  (cd groupA;/usr/bin/time -v taskset -c 0-$(($t-1)) $kmc_dump A_specific A_specific.txt &> dump.log)  (cd groupB;/usr/bin/time -v taskset -c 0-$(($t-1)) ./count.sh $kmc $k $t &> count.log)  (cd groupB;/usr/bin/time -v taskset -c 0-$(($t-1)) $kmc_tools -hp -t$t complex complex.op &> intersection.log)  (cd groupB;/usr/bin/time -v taskset -c 0-$(($t-1)) $kmc_tools -hp -t$t simple B_intersection ../ALL intersect inter2 -ocmax &> ALL_intersect.log)  (cd groupB;/usr/bin/time -v taskset -c 0-$(($t-1)) $kmc_tools -hp -t$t simple inter2 B_intersection counters_subtract invalid &> counters_subtract.log)  (cd groupB;/usr/bin/time -v taskset -c 0-$(($t-1)) $kmc_tools -hp -t$t simple B_intersection invalid kmers_subtract B_specific &> kmers_subtract.log)  (cd groupB;/usr/bin/time -v taskset -c 0-$(($t-1)) $kmc_dump B_specific B_specific.txt &> dump.log) |
| --- |

1.2.2 File *groupA/complex.op*

| INPUT:  set_SRR3050947_sorted = ./SRR3050947.fasta  set_SRR3050849_sorted = ./SRR3050849.fasta  set_SRR3050911_sorted = ./SRR3050911.fasta  set_SRR3050953_sorted = ./SRR3050953.fasta  set_SRR3050956_sorted = ./SRR3050956.fasta  set_SRR3050988_sorted = ./SRR3050988.fasta  set_SRR3050916_sorted = ./SRR3050916.fasta  set_SRR3051015_sorted = ./SRR3051015.fasta  set_SRR3050919_sorted = ./SRR3050919.fasta  set_SRR3050851_sorted = ./SRR3050851.fasta  set_SRR3050918_sorted = ./SRR3050918.fasta  set_SRR3050965_sorted = ./SRR3050965.fasta  set_SRR3050978_sorted = ./SRR3050978.fasta  set_SRR3050937_sorted = ./SRR3050937.fasta  set_SRR3050886_sorted = ./SRR3050886.fasta  set_SRR3050845_sorted = ./SRR3050845.fasta  set_SRR3050966_sorted = ./SRR3050966.fasta  set_SRR3050850_sorted = ./SRR3050850.fasta  set_SRR3050973_sorted = ./SRR3050973.fasta  set_SRR3050927_sorted = ./SRR3050927.fasta  set_SRR3050889_sorted = ./SRR3050889.fasta  set_SRR3051028_sorted = ./SRR3051028.fasta  set_SRR3050847_sorted = ./SRR3050847.fasta  set_SRR3050858_sorted = ./SRR3050858.fasta  set_SRR3050859_sorted = ./SRR3050859.fasta  set_SRR3050920_sorted = ./SRR3050920.fasta  set_SRR3050867_sorted = ./SRR3050867.fasta  set_SRR3050908_sorted = ./SRR3050908.fasta  set_SRR3050881_sorted = ./SRR3050881.fasta  set_SRR3051006_sorted = ./SRR3051006.fasta  set_SRR3050900_sorted = ./SRR3050900.fasta  set_SRR3050899_sorted = ./SRR3050899.fasta  set_SRR3050940_sorted = ./SRR3050940.fasta  set_SRR3050848_sorted = ./SRR3050848.fasta  set_SRR3050866_sorted = ./SRR3050866.fasta  set_SRR3050934_sorted = ./SRR3050934.fasta  set_SRR3050984_sorted = ./SRR3050984.fasta  set_SRR3051001_sorted = ./SRR3051001.fasta  set_SRR3050879_sorted = ./SRR3050879.fasta  set_SRR3050929_sorted = ./SRR3050929.fasta  set_SRR3050907_sorted = ./SRR3050907.fasta  set_SRR3050895_sorted = ./SRR3050895.fasta  set_SRR3050958_sorted = ./SRR3050958.fasta  set_SRR3051035_sorted = ./SRR3051035.fasta  set_SRR3050931_sorted = ./SRR3050931.fasta  set_SRR3050975_sorted = ./SRR3050975.fasta  set_SRR3050922_sorted = ./SRR3050922.fasta  set_SRR3050961_sorted = ./SRR3050961.fasta  set_SRR3050933_sorted = ./SRR3050933.fasta  set_SRR3051026_sorted = ./SRR3051026.fasta  set_SRR3050982_sorted = ./SRR3050982.fasta  set_SRR3050875_sorted = ./SRR3050875.fasta  OUTPUT:  A_intersection = set_SRR3050947_sorted * sum set_SRR3050849_sorted * sum set_SRR3050911_sorted * sum set_SRR3050953_sorted * sum set_SRR3050956_sorted * sum set_SRR3050988_sorted * sum set_SRR3050916_sorted * sum set_SRR3051015_sorted * sum set_SRR3050919_sorted * sum set_SRR3050851_sorted * sum set_SRR3050918_sorted * sum set_SRR3050965_sorted * sum set_SRR3050978_sorted * sum set_SRR3050937_sorted * sum set_SRR3050886_sorted * sum set_SRR3050845_sorted * sum set_SRR3050966_sorted * sum set_SRR3050850_sorted * sum set_SRR3050973_sorted * sum set_SRR3050927_sorted * sum set_SRR3050889_sorted * sum set_SRR3051028_sorted * sum set_SRR3050847_sorted * sum set_SRR3050858_sorted * sum set_SRR3050859_sorted * sum set_SRR3050920_sorted * sum set_SRR3050867_sorted * sum set_SRR3050908_sorted * sum set_SRR3050881_sorted * sum set_SRR3051006_sorted * sum set_SRR3050900_sorted * sum set_SRR3050899_sorted * sum set_SRR3050940_sorted * sum set_SRR3050848_sorted * sum set_SRR3050866_sorted * sum set_SRR3050934_sorted * sum set_SRR3050984_sorted * sum set_SRR3051001_sorted * sum set_SRR3050879_sorted * sum set_SRR3050929_sorted * sum set_SRR3050907_sorted * sum set_SRR3050895_sorted * sum set_SRR3050958_sorted * sum set_SRR3051035_sorted * sum set_SRR3050931_sorted * sum set_SRR3050975_sorted * sum set_SRR3050922_sorted * sum set_SRR3050961_sorted * sum set_SRR3050933_sorted * sum set_SRR3051026_sorted * sum set_SRR3050982_sorted * sum set_SRR3050875_sorted |
| --- |

1.2.3 File *groupA/count.sh*

| #!/bin/bash  function run_command  {  command=$1  echo $command  eval $command  }  kmc=$1  k=$2  t=$3  mkdir -p tmp  for file in `cat ../GroupA.txt \| sed 's/\r//g'`  do  array=(${file//// })  run_command "$kmc -k$k -ci2 -cs16777215 -fm -t$t ${file} ${array[-1]} tmp"  done |
| --- |

2 GenomeTester4 (version 4.0) running command to identify unique k-mers

2.1 Files in GenomeTester4 program files folder

| Name | Type | Description |
| --- | --- | --- |
| GroupA.txt | Text | All group A [high-throughput](javascript:;) [sequencing](javascript:;) file paths |
| GroupB.txt | Text | All group B [high-throughput](javascript:;) [sequencing](javascript:;) file paths |
| run.sh | Linux shell script | A script to run this program |
| count.sh | Linux shell script | A script to count k-mer |
| MakeIntersection.pl | Perl script | Operation for intersection |
| MakeUnion.pl | Perl script | Operation for union |

2.2 Files

1.2.1 File *run.sh*

| #!/bin/bash  k=31  file_setA=""  file_setB=""  for file in `cat ./GroupA.txt \| sed 's/\r//g'`  do  array=(${file//// })  file_setA=$file_setA" ./GroupA/${array[-1]}_${k}.list"  done  for file in `cat ./GroupB.txt \| sed 's/\r//g'`  do  array=(${file//// })  file_setB=$file_setB" ./GroupB/${array[-1]}_${k}.list"  done  /usr/bin/time -v -o log_count_time.txt ./count.sh  echo "./MakeUnion.pl${file_setA}${file_setB}"  /usr/bin/time -v -o log_union_time.txt ./MakeUnion.pl${file_setA}${file_setB}  mv union_${k}_union.list all_${k}_union.list  rm -rf union*  echo "./MakeIntersection.pl${file_setA}"  /usr/bin/time -v -o log_intrsec_A_time.txt ./MakeIntersection.pl${file_setA}  mv intrs_${k}_intrsec.list A_${k}_intrsec.list  rm -rf intrs*  echo "./MakeIntersection.pl${file_setB}"  /usr/bin/time -v -o log_intrsec_B_time.txt ./MakeIntersection.pl${file_setB}  mv intrs_${k}_intrsec.list B_${k}_intrsec.list  rm -rf intrs*  /usr/bin/time -v -o log_diff_union_A_time.txt glistcompare A_${k}_intrsec.list all_${k}_union.list -du -o A_specific  /usr/bin/time -v -o log_diff_union_B_time.txt glistcompare B_${k}_intrsec.list all_${k}_union.list -du -o B_specific |
| --- |

1.2.2 File *count.sh*

| #!/bin/bash  t=20  k=31  mkdir -p GroupA  mkdir -p GroupB  for file in `cat ./GroupA.txt \| sed 's/\r//g'`  do  array=(${file//// })  glistmaker ${file} -w ${k} --num_threads ${t} -c 2 -o ./GroupA/${array[-1]}  done  for file in `cat ./GroupB.txt \| sed 's/\r//g'`  do  array=(${file//// })  glistmaker ${file} -w ${k} --num_threads ${t} -c 2 -o ./GroupB/${array[-1]}  done |
| --- |

3 CAP3

3.1 Command example

*cap3 speicific-40mers.fa -i 30 -j 31 -o 18 -s 300*

3.2 Parameters

| Short option | Description |
| --- | --- |
| -i | specify segment pair score cutoff |
| -j | specify chain score cutoff |
| -o | specify overlap length cutoff |
| -s | specify overlap similarity score cutoff |
